# Novel HER2 Aptamer Selectively Delivers Cytotoxic Drug to HER2-positive *Breast* Cancer Cells *in Vitro*

**DOI:** 10.1186/1479-5876-10-148

**Published:** 2012-07-20

**Authors:** Zhe Liu, Jin-Hong Duan, Yong-Mei Song, Jie Ma, Feng-Dan Wang, Xin Lu, Xian-Da Yang

**Affiliations:** 1 Institute of Basic Medical Sciences, Chinese Academy of Medical Sciences & Peking Union Medical College, Beijing 100005, China; 2 Cancer Institute & Hospital, Chinese Academy of Medical Sciences & Peking Union Medical College, Beijing 100021, China; 3 Peking Union Medical College Hospital, Chinese Academy of Medical Sciences & Peking Union Medical College, Beijing 100073, China

**Keywords:** Aptamer, HER2, Breast cancer, Tumor targeted therapy

## Abstract

**Background:**

Aptamer-based tumor targeted drug delivery system is a promising approach that may increase the efficacy of chemotherapy and reduce the related toxicity. HER2 protein is an attractive target for tumor-specific drug delivery because of its overexpression in multiple malignancies, including breast, gastric, ovarian, and lung cancers.

**Methods:**

In this paper, we developed a new HER2 aptamer (HB5) by using systematic evolution of ligands by exponential enrichment technology (SELEX) and exploited its role as a targeting ligand for delivering doxorubicin (Dox) to breast cancer cells *in vitro*.

**Results:**

The selected *aptamer* was an 86-nucleotide DNA molecule that bound to an epitope peptide of HER2 with a *K*_d_ of 18.9 nM. The aptamer also bound to the extracellular domain (ECD) of HER2 protein *with a K*_*d*_*of 316 nM*, and had minimal cross reactivity to albumin or trypsin. In addition, the aptamer was found to preferentially bind to HER2-positive but not HER2-negative breast cancer cells. An aptamer-doxorubicin complex (Apt-Dox) was formulated by intercalating Dox into the DNA structure of HB5. The Apt-Dox complex could selectively deliver Dox to HER2-positive breast cancer cells while reducing the drug intake by HER2-negative cells *in vitro*. Moreover, Apt-Dox retained the cytotoxicity of Dox against HER2-positive *breast cancer* cells, but reduced the cytotoxicity to HER2-negative cells.

**Conclusions:**

The results suggest that the selected HER2 aptamer may have application potentials in targeted therapy against HER2-positive *breast cancer cells*.

## Background

Breast cancer is the most common malignancy among women worldwide. The overexpression of HER2 (human epithelial growth factor receptor 2) is found in approximately 20-30% of breast cancer [1]. HER2-positive breast cancer is associated with more malignant behaviors, including increased invasiveness, higher recurrence, and reduced overall survival [2-4], comparing to other types of breast cancer. Moreover, more than half of the HER2-positive women are ER/PR-negative, suggesting that a fair proportion of HER2-positive breast cancers do not respond well to endocrine therapies [5,6]. For those patients only accepting the conventional treatments of surgical resection followed by chemo- and endocrine therapies, the median survival time of HER2-positive patients was about half of that of HER2-negative cases [7]. In view of the above situation, the development of novel therapy for HER2-positive breast cancer, such as tumor-targeted therapy, is an inevitable trend. Targeted therapy is characterized by selective killing of tumor cells with minimal influence to normal cells, and may improve therapeutic efficacy and reduce adverse effect. Trastuzumab (Herceptin), a humanized HER2 monoclonal antibody, has been approved as a first line targeted treatment of HER2-positive metastatic breast cancer. However, drug resistance is developed rapidly with trastuzumab treatment in virtually all patients [2,3,8-11]. Therefore, it is necessary to develop novel targeting therapeutic strategies for treatment of HER2-positive breast cancer.

Tumor-targeted therapy requires ligands that can bind to cancer cells. In addition to antibodies, other types of novel tumor-targeting ligands have emerged in the past decades [12]. One new class of ligands are aptamers, which are single-stranded oligonucleotides that may serve as targeting molecules in therapeutic and diagnostic applications [12]. Aptamers have certain advantages as ligands, such as high affinity, excellent specificity, and low immunogenicity. Aptamers are also easy to synthesize and modify chemically [13]. Based on these advantages, aptamers have broad prospect in therapeutic applications. The first aptamer drug, Mucagen, was approved for clinical use years ago. Other aptamer-based agents are being actively tested in clinical trials [14,15]. Moreover, aptamers could also be employed as tumor-targeting ligands to significantly enhance the treatment outcome in animal tumor models [16-19]. A specially designed aptamer–siRNA chimera that significantly enhanced the inhibition of prostate cancer in mice when administered systemically [19]. It was also reported that aptamer-conjugated to docetaxel-containing nanoparticles could increase the cancer-specific cytotoxicity both *in vitro* and *in vivo *[18]. These studies suggest that aptamers can serve as excellent tumor-targeting ligands in targeted therapeutic systems.

So far, however, no aptamer has been explored as tumor-targeting ligand for delivering chemotherapeutic agent to HER2-positive breast cancer, and there are limited published studies on HER2 aptamers [20,21]. In the present study, we developed a new DNA aptamer (HB5), which was found capable of binding to both the HER2 protein and the HER2-positive breast cancer cells. To evaluate whether the aptamer could be employed to selectively carry cytotoxic drug to HER2-positive breast cancer cells, we also constructed an aptamer-doxirubicin complex (Apt-Dox). We now report that Apt-Dox can selectively deliver doxorubicin to HER2-positive breast cancer cells *in vitro*.

## Methods

### Cell lines and cultures

The Cell lines, SK-BR-3 (human breast cancer), MDA-MB-231 (human breast cancer), MCF-7 (human breast adenocarcinoma) were obtained from the Cell Resource Center of Chinese Academy of Medical Sciences (Beijing, China). All cell lines were incubated in DMEM medium supplemented with 100 U/mL penicillin, 100 μg/mL streptomycin and 10% fetal bovine serum (Gibico) in 5% CO2 and humid atmosphere at 37°C. All experiments were performed on cells in the exponential growth phase.

### Random DNA library and primers

A starting ssDNA library consisted of 86 nucleotides, containing a 40-base random region flanked by two fixed sequences for the amplification reaction. The library sequence was 5’-AACCGCCCAAATCCCTAAGAGTC-N40-CACAGACACACTACACACGCACA-3’. A FITC- labeled 5’ Primer P1 (5’-FITC-AACCGCCCAAATCCCTAAGAGTC-3’) was used in the PCR to monitor the binding of aptamer with target agents during the selection process. A biotinylated 3’ primer P2 (5’-biotin-TGTGCGTGTGTAGTGTGTCTGTG-3’) was used to amplify double strand DNA (dsDNA) and enable this double labeled dsDNA binding to streptavidin-coated magnetic beads. The FITC-conjugated sense single-strand DNA (ssDNA) was separated from the biotin- and FITC- labeled dsDNA by denaturing in alkaline condition (0.1 M NaOH) and applying a magnetic field. The FITC-labeled ssDNA were used for aptamer selection. Unlabeled P1 and P2 primers were used for cloning the enriched library after the end of selection. All primers and the initial random DNA library were synthesized and purified by Invitrogen China. Streptavidin-coated magnetic beads were purchased from Promega (USA).

### Targets immobilization

HER2 Peptide of at least 95% purity was synthesized by SBS Genetech (Beijing, China). Magnetic monodispersed microspheres embedded in SiO2 (Affimag SLE, 3 μm- 4 μm) were purchased from BaseLine ChromTech (Tianjin, China). 1-Ethyl-3-(3-dimethyllaminopropyl) carbodiimide hydrochloride (EDC) was purchased from Sigma. Bovine serum albumin (BSA) and trypsin were purchased from Amresco Inc. (USA). The extracellular domain of HER2 protein (HER2 ECD) was purchased from Sino Biological Inc. (Beijing, China).

A peptide from the juxtamembrane region of HER2 ECD was used as the target for selection, with the sequence of INCTHSCVDLDDKGCPAEQR. The HER2 peptide was immobilized to carboxylated magnetic beads through cross-linking of –COOH and –NH2. The carboxylated SLE beads (1x10^5^, 200 μL) were washed twice with 200 μL of PBS. The beads were resuspended in 200 μL deionized water dissolving with HER2 peptide (2 μg) and EDC (40 mM), and then incubated at room temperature with gentle stirring for 2 h. The beads were then washed for three times with PBS, and stored at 4°C in PBS. The same method was utilized to conjugate the beads with other substances, including HER2 ECD protein, bovine serum albumin (BSA), trypsin and HER2 ECD, for specificity assays of the selected aptamer.

### SELEX process

To reduce background interference, 0.1 mg/mL salmon sperm DNA and 1 mg/mL of BSA were added to the binding buffer (Hanks buffer). The procedures of selection were as follows: in the initial selection round, the ssDNA pool (200 pmol) was first heated at 95°C for 5 min and then cooled immediately on ice for 15 min. The HER2-peptide coated beads were suspended in 200 μL of binding buffer containing 200 pmol of random ssDNA. After incubating the mixture at 37°C for 30 min with gentle shaking, the unbound oligonucleotides were removed by washing four times with 500 μL of binding buffer. Subsequently, target-binding oligonucleotides on beads were amplified by PCR with FITC- or biotin-labeled primers (25 cycles of 40 s at 94°C, 30 s at 65°C, 40 s at 72°C, followed by 10 min at 72°C, the Taq polymerase and dNTPs were obtained from Takara). The selected sense ssDNA were separated from the biotinylated antisense ssDNA strand by streptavidin-coated magnetic beads and used for the next round of SELEX. After multiple rounds of selection, the selected ssDNA pool was PCR-amplified using unmodified primers and cloned into Escherichia coli with the TA cloning kit for DNA sequencing.

### Flow cytometric analysis

To monitor the enrichment of aptamer pool each round, the FITC-labeled ssDNA pool were incubated with HER2-peptide coated magnetic beads in 200 μL of selection buffer containing 10% FBS at 37°C for 30 min. The beads were washed twice with 0.5 mL of binding buffer, suspended in 0.2 ml of binding buffer, and then analyzed by Flow cytometry (Accuri® C6 Flow Cytometer, BD). Peptide-coated beads stained with FITC-labeled random ssDNA were used as control.

To evaluate the binding specificity of the aptamers, FITC-labeled aptamers were separately incubated with HER2 ECD, BSA, or trypsin-coated magnetic beads. The beads were washed twice with 0.5 mL of binding buffer, suspended in 0.2 mL of binding buffer, and analyzed by Flow Cytometry. For assessing aptamer binding on cells, the cells were scraped off the culture bottle and washed twice with Hanks buffer. The FITC-labeled aptamers were incubated separately with 5 × 10^5^ of SK-BR-3, MDA-MB-231, or MCF-7 in binding buffer at 37°C for 30 min. Cells were then washed twice with Hanks buffer and analyzed by flow cytometry.

To evaluate the binding affinity of the aptamer to HER2 structure, we incubated HER2-peptide coated magnetic beads with varying concentrations of the FITC-labeled aptamer HB5 in 200 μL binding buffer at 37°C for 30 min. The beads were washed twice with 0.5 mL of binding buffer, suspended in 0.2 mL of binding buffer, and then analyzed by Flow Cytometry. The peptide-coated beads stained with FITC-labeled random ssDNA were used as a control. All of the experiments for binding assay were repeated for at least three times. The mean fluorescence intensity of the aptamer bound to the target was used to calculate the specific binding by subtracting the mean fluorescence intensity of nonspecific binding from unselected library DNA. The equilibrium dissociation constant (*K*_d_) of the aptamer was obtained by fitting the dependence of fluorescence intensity of specific binding on the concentration of the aptamers to the equation: Y = B max X/ ( *K*_d_ + X) [22].

### Apt-Dox complex

The aptamer were first heated at 95°C for 5 min and then cooled immediately on ice for 15 min. Next, a fixed concentration of Dox (3 nM) was incubated for 1 h with various concentrations of aptamer HB5, at aptamer/Dox molar ratios of 1, 0.1, 0.01, 0.003, 0.0001, and 0, respectively. The fluorescence spectrum of doxorubicin was examined in 96-well black plate by a Synergy4 analyzer (λ_Ex_ = 488 nm, λ_Em_ = 520–720 nm).

### Cellular uptake studies

The cellular uptake of Apt-Dox or free Dox by cells was studied by confocal microscopy (Perkin Elmer Ultraview, US) and flow cytometry. Cells were allowed to adhere to a glass cover slip for 24 h. The cells were then incubated with 1.5 μM of Dox or Apt-Dox complex for 2 h at 37°C. After washing twice with Hanks buffer, the cells were fixed with 4% formaldehyde for 10 min and analyzed by confocal fluorescence scanning microscopy.

For flow cytometry analysis, cells were scraped off from the culture bottle and washed twice with Hanks buffer. The cells were incubated with 1.5 μM Dox or Apt-Dox complex for 4 h at 37°C, and washed twice with Hanks buffer. The cells were then fixed with 4% formaldehyde for 10 min and analyzed by Flow Cytometry.

### *In vitro* cytotoxicity assays

To evaluate the cytotoxicity of Apt-Dox or Dox against SK-BR-3 and MDA-MB-231 cells, both cell lines were first grown in 96-well plates, and then co-incubated with Apt-Dox, Dox, or aptamer separately at the concentration of 2 μM for 4 h at 37°C. The cells were washed with Hanks buffer twice, and cultured for a further 40 h. Afterwards, MTS viability assay was performed according to the standard protocol outlined by the manufacture (Promega, US).

### Statistics

Statistical analysis was performed with the statistical SPSS 13.0 software. The nonparametric test was used to calculate the probability of significant differences among the groups. Statistical significance was defined as p < 0.05.

## Results

### Aptamer selection

**Figure 1:**
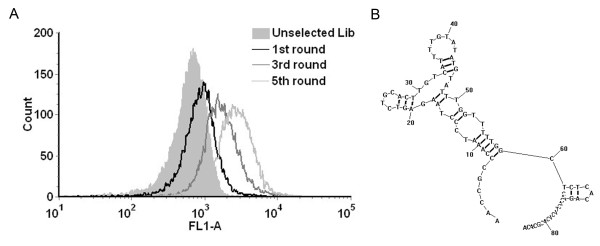
**Aptamer selection. ** (**A**) Flow cytometry profiles monitoring the aptamer enrichment during the selection process. From left to right, starting random DNA pool (solid histogram), 1^st^ round (black line), 3^rd^ round (grey line), and 5^th^ round (silver line). *(****B****) The predicted secondary structure of the aptamer HB5*.

In this study, the HER2 aptamers were developed using a standard SELEX technique and a HER2 epitope peptide as the target. The peptide was conjugated covalently to SLE beads via EDC-mediated reaction. To monitor the enrichment of HER2 aptamers during each round of selection, the handled beads were incubated with FITC-labeled ssDNA pool for flow cytometry assay. Compared with the initial random DNA library, increasing fluorescent intensities were observed, suggesting that more ssDNA bound to the peptide-coated beads with each round of selection (Figure 1A). The enriched HER2 aptamers were subsequently cloned, and 96 clones were analyzed for further characterization. Among these clones, one aptamer termed HB5 showed relatively high binding capacity to the target HER2 peptide. The primary sequence of the aptamer HB5 is 5’-AACCGCCCAAATCCCTAAGAGTCTGCACTTGTCATTTTGTATATGTATTTGGTTTTTGGCTCTCACAGACACACTACACACGCACA-3’. The predicted secondary structure of HB5 was shown in Figure 1B.

### Binding property of the aptamer HB5

**Figure 2:**
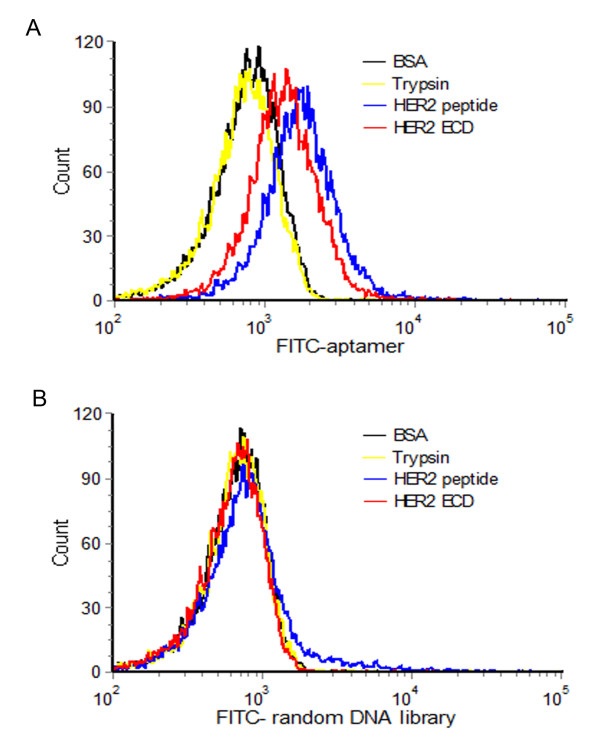
**Flow cytometric evaluations of aptamer HB5’s binding property.** Beads coated with HER2 peptide (blue lines), HER2 ECD protein (red lines), BSA (black lines), or trypsin (yellow lines) were incubated separately with either FITC-labeled aptamer (**A**) or FITC-labeled random DNA (**B**), and analyzed by flow cytometry.

The aptamer HB5 was selected to bind the target HER2 peptide. It is important for the aptamer to also recognize the extracellular domain (ECD) of HER2 protein, which is the exposed HER2 structure on tumor cells. We therefore evaluated the binding of the atpamer to the HER2 ECD. The HER2 ECD protein was covalently conjugated to magnetic beads, incubated with FITC-labeled HB5, and analyzed by flow cytometry. FITC-labeled initial random DNA library was used as control. As shown in Figure 2 (red lines), the fluorescent signal of HB5 increased significantly over the control. The data indicated that, in addition to binding the target HER2-peptide, the aptamer HB5 could also bind to the HER2 ECD protein.

To evaluate whether the aptamer HB5 had a relatively preferred binding to the HER2 structure, *we directly compared, under the same experimental conditions, the bindings of the aptamer to HER2 peptide, HER2 ECD protein, albumin (BSA), or trypsin. Albumin was tested because it is the most abundant protein in blood; and trypsin was commonly used for testing the binding specificity of the aptamers. The results are presented in Figure*2*. While aptamer HB5 showed relatively strong bindings to HER2 peptide and HER2 ECD protein, its binding to BSA or trypsin was weak (*Figure 2*A). In addition, random DNA showed no binding preference to all four structures (*Figure 2*B).* The data suggested that the aptamer HB5 had a targeting preference towards HER2, and tended not to bind the other proteins such as BSA or trypsin.

**Figure 3:**
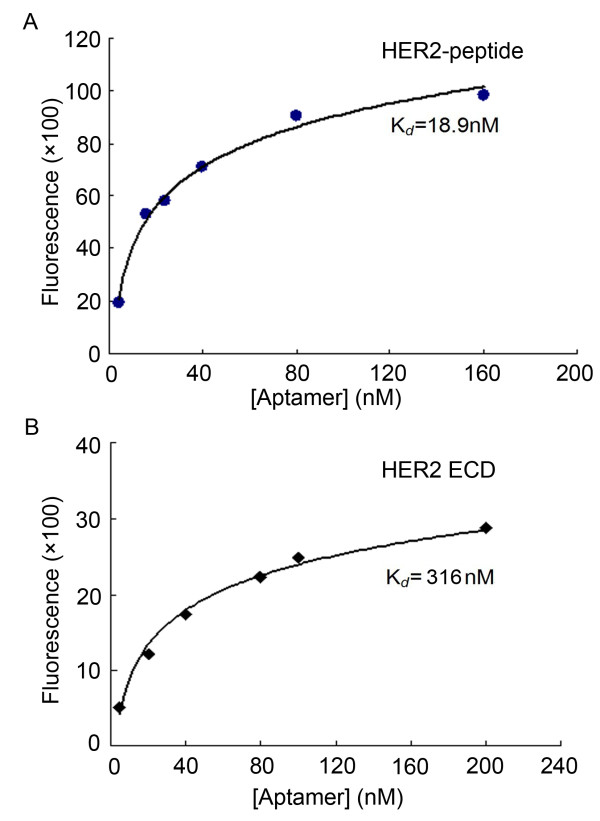
**Quantitative assay of HB5’s binding affinity.** Beads coated with HER2 peptide (**A**) *or HER2 ECD protein (****B****) * were incubated with varying concentrations of FITC-labeled aptamers. The mean fluorescence intensity of the unselected DNA library (background binding) at each concentration was subtracted from the mean fluorescence intensity of the corresponding aptamer. The actual fluorescence intensity was fitted into Sigmaplot to determine the apparent Kd.

To quantitatively evaluate the HER2 binding affinity of the aptamer HB5, HER2 peptide-coated beads were incubated with increasing concentrations of FITC-labeled HB5 and analyzed by flow cytometry. Using a non-linear regression analysis, the *K*_d_ of the aptamer for binding with the HER2 peptide was estimated to be 18.9 nM (Figure 3A). *It is also important to quantify the affinity of the aptamer for recognizing the extracellular domain (ECD) of HER2 protein, which is the exposed HER2 structure on tumor cells. Similar experiments were conducted using beads coated with HER2 ECD protein, and the K*_*d*_*of the aptamer for binding with the HER2 ECD was estimated to be 316 nM (*Figure 3*B).*

### Aptamer HB5 selectively recognized HER2-positive breast cancer cells

**Figure 4:**
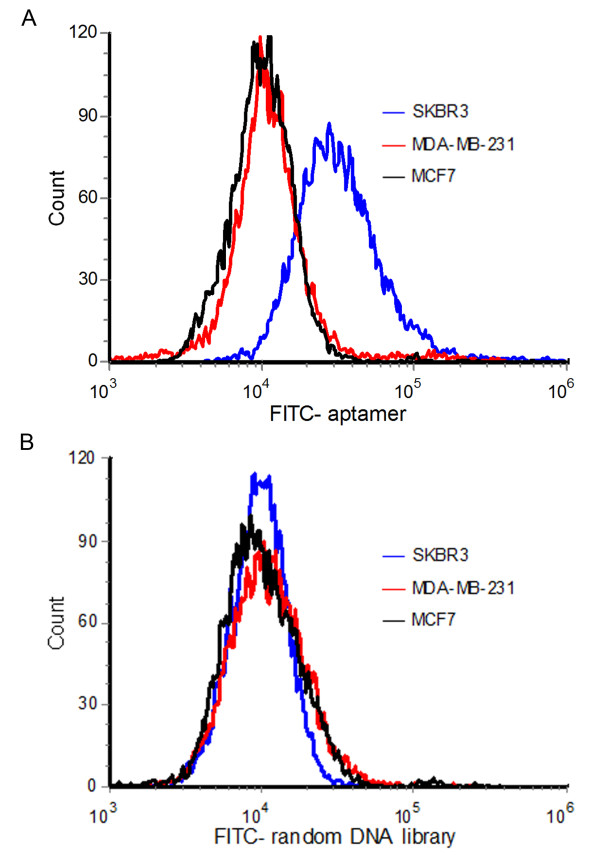
**Flow cytometric profiles of HB5’s bindings to HER2-positive (SKBR3) or HER2-negative (MDA-MB-231 and MCF7) breast cancer cells.** SKBR3 (blue lines), MDA-MB-231 (red lines), or MCF7 (black lines) cells were incubated separately with either FITC-labeled aptamer (**A**) or FITC-labeled random DNA (**B**), and analyzed by flow cytometry.

Although the aptamer HB5 demonstrated good binding profiles for the HER2 peptide and the HER2 ECD protein, it is still unknown whether the aptamer would bind to the HER2-positive breast cancer cells. To address this issue, *we directly compared the bindings of the aptamer to either the HER2-positive (SK-BR-3), or the HER2-negative breast cancer cells (MDA-MB-231 and MCF-7) *[23]*under the same experimental conditions.* The HER2 expressions in these breast cancer cell lines had been extensively analyzed by prior studies with mRNA or Western assays [24-27]. FITC-labeled HB5 was incubated with the cells, which were later analyzed by flow cytometry. The cells incubated with FITC-labeled random DNA were used as the control. *As presented in Figure*4*, while aptamer HB5 showed relative strong binding to HER2-positive breast cancer cells (SK-BR-3), the bindings to HER2-negative cells (MDA-MB-231 and MCF-7) were weak (*Figure 4*A). In addition, random DNA showed no binding preference to all the cell lines (*Figure 4*B).* The results indicated that the aptamer HB5 could preferentially bind to HER2-positive breast cancer cells, possibly by recognizing the HER2 structure on the surface of these cells.

### Formation of aptamer-doxorubicin complex

**Figure 5:**
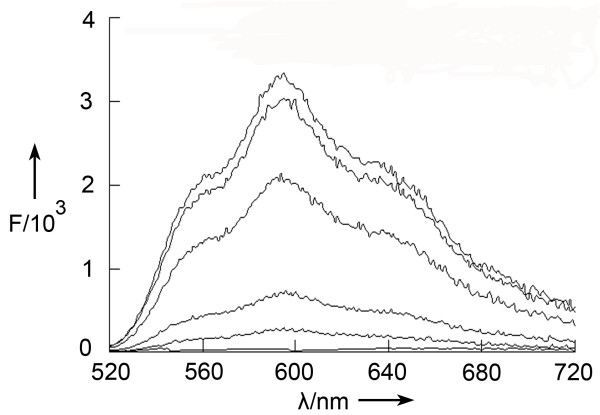
**Fluorescence spectra assay of Apt-Dox.** Doxorubicin solution mixed with increasing molar ratios of the HB5 aptamer (from top to bottom: 0, 0.0001, 0.003, 0.03, 0.1, and 1).

To explore whether the aptamer HB5 could be employed to selectively carry cytotoxic agents to HER2-positive breast cancercells, we next constructed an aptamer-doxorubicin complex (Apt-Dox), utilizing the fact that doxorubicin tended to intercalate into DNA structures. The first question here was whether Dox indeed intercalated into the DNA structure of the aptamer HB5. It is well known that Dox emits a fluorescence that will be quenched after intercalation into DNA structure [28]. This feature was used in this study to evaluate the formation of the Apt-Dox complex. Specifically, a fixed concentration of Dox was mixed with HB5 aptamers of increasing molar ratios. The mixtures were later analyzed by fluorescence spectroscopy. As shown in Figure 5, the native fluorescence spectrum of Dox decreased with increasing concentrations of the aptamer. The fluorescence of Dox was almost completely quenched when the Apt/Dox molar ratio gradually increased to 0.1. The results suggested that most Dox intercalated into the DNA structure of the aptamer after the Apt/Dox molar ratio increased to 0.1 or above.

### The Apt-Dox complex was selectively uptaken into HER2-positive breast cancer cells

**Figure 6:**
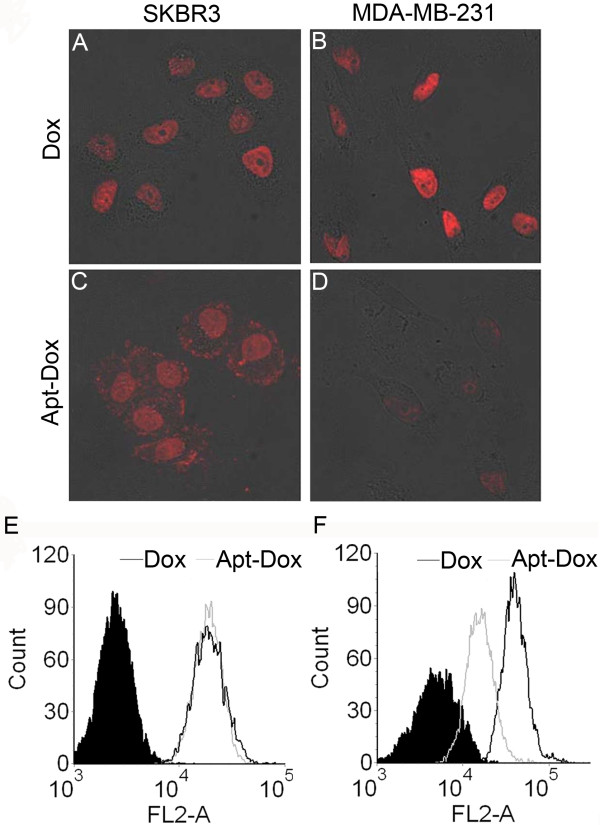
**Evaluation of cellular uptake of Dox.** Confocal microscopy images of HER2-positive SK-BR-3 cells and HER2-negative MDA-MB-231 cells treated with free Dox (**A**&**B**) or Apt-Dox (**C**&**D**). Flow cytometry profiles of HER2-positive SK-BR-3 cells (**E**) and HER2-negative MDA-MB-231 cells (**F**) after treatments with free Dox (black lines) or Apt-Dox (grey lines). The filled histograms are the control signals generated by untreated cells.

To evaluate the feasibility of the Apt-Dox complex as a tumor-targeted drug delivery system, we performed *in vitro* drug uptake studies with both HER2-positive and -negative breast cancer cells. The HER2-positive breast cancer cells used here were SK-BR-3 cells, which abundantly express HER2 protein on their plasma membrane [1,26]. The HER2-negative cells were the MDA-MB-231 cells, which do not express any detectable level of HER2 protein [29,30]. Confocal microscopy was employed to evaluate the fluorescence emitted by Dox and the uptake of the drug by the two types of cells. While free Dox was taken up by both cell lines (Figure 6A&B), the Apt-Dox was mainly uptaken by HER2-positive cells (Figure 6C). The results suggested that Apt-Dox could discriminate efficiently between HER2-positive and -negative cells, and that the aptamer HB5 possibly retained HER2-binding ability while carrying the Dox within its DNA structure. It should be noted that the Apt-Dox produced very weak staining in MDA-MB-231 cells (Figure 6D), a result consistent with the lack of HER2 expression in these cells. Overall, the data indicated that the Apt-Dox conjugate could selectively enter HER2-positive breast cancer cells, while free Dox could not differentiate between HER2-positive and -negative cells. Interestingly, in HER2-positive SK-BR-3 breast cancer cells, the Apt-Dox stained both the nuclei and the cytoplasm (Figure 6C), while the free Dox exclusively stained the cell nuclei (Figure 6A). This suggested that the uptake mechanisms of the free DOX and the Apt-Dox complex might be different.

Next we performed flow cytometry experiments to further evaluate the uptake of Apt-Dox and free Dox by HER2-positive and -negative breast cancer cells (Figure 6E&F). For HER2-positive SK-BR-3 cells, the fluorescent signals from free Dox or Apt-Dox were similar (Figure 6E); whereas for HER2-negative MDA-MB-231 cells, the fluorescent signal from Apt-Dox was lower than that from free Dox (Figure 6F). Taken together, both the confocal and flow cytometry studies suggested that Apt-Dox conjugate mainly entered HER2-positive breast cancer cells, while free Dox entered both HER2-positive and -negative cells.

### The Apt-Dox complex selectively reduced cytotoxity to HER2-negative breast cancer cells

**Figure 7:**
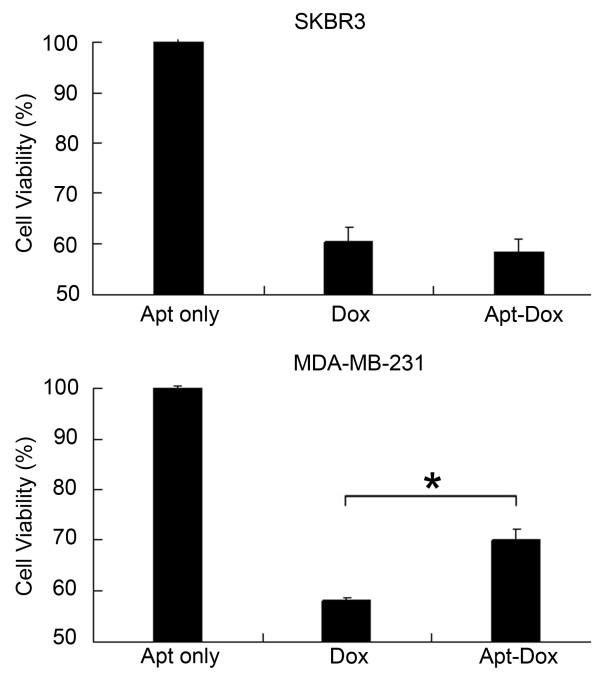
**Cytotoxicity assays of Dox and Apt-Dox.** The HER2-positive SK-BR-3 cells and the HER2-negative MDA-MB-231 cells were treated with aptamer, Dox, or Apt-Dox. After 40 h of further incubation, the cells were evaluated with standard MTS assay (mean ± SD, n = 6). The star indicates a statistically significant difference between the Dox and the Apt-Dox groups (p < 0.05).

To examine whether the selective delivery of Apt-Dox to HER2-positive breast cancer cells would result in targeted cytotoxicity, we compared the cytotoxic efficacies of free Dox and Apt-Dox on HER2-positive and -negative cells *in vitro* with MTS assay. The data showed that while free Dox produced similar cytotoxicity against HER2-positive and -negative breast cancer cells, Apt-Dox generated a moderately reduced cytotoxicity against the HER2-negative cells (Figure 7, *p* < 0.05). The aptamer itself had no inherent cytotoxicity to HER2-positive or -negative cells. Our data suggested that the HER2 aptamer could selectively deliver Dox to HER2-positive breast cancer cells and might potentially reduce the adverse effects of the drug to HER2-negative cells.

## Discussion

HER2 over-expression is found not only in 20–30% of breast cancers, but also in gastric, ovarian, bladder, and lung cancers [22,31-36]. Therefore, HER2 may serve as a potential target for targeted tumor therapy against many malignancies. Aptamers can bind to target with high affinity and specificity, and may be employed as targeting ligand in selective drug delivery system. Here in this study, using the SELEX technique with a HER2 peptide as the target, we developed a novel HER2 aptamer (HB5) with a *K*_d_ of 18.9 nM for binding with the target peptide (Figure 1 &3A). In addition to binding the HER2 peptide, HB5 could also bind to HER2 ECD protein *with a K*_*d*_*of 316 nM* (Figure 2 &3B), and with minimal cross-reactivity to trypsin and albumin (Figure 2), suggesting that the aptamer had a binding preference for HER2 structures. Moreover, the aptamer HB5 could selectively bind to HER2-positive breast cancer cells with weak cross-reaction to HER2-negative cells (Figure 4). An aptamer-doxorubicin complex (Apt-Dox) was formed by intercalating Dox into the DNA structure of the aptamer (Figure 5). While retaining the efficacy of doxorubicin against HER2-positive breast cancer cells, the Apt-Dox complex reduced the drug intake and the toxicity to HER2-negative cells (FigureS 6&7). The results suggested that Apt-Dox could discern target and non-target cells, and that HER2 aptamer might serve as targeting ligand for selective drug delivery to HER2-positive *breast cancer* cells.

Monoclonal antibodies have been the main approach for HER2-targeted cancer therapy, and trastuzumab (Herceptin), a humanized HER2 monoclonal antibody, has been approved for treatment of HER2-positive breast cancer. However, drug resistance is commonly and rapidly developed with trastuzumab treatment [2,3,8-11], making it necessary to explore other HER2-targeting therapeutic strategies. Recent studies have identified some RNA or DNA HER2 aptamers [20,21]. Kim *et al.* developed an RNA aptamer using HER2 protein as the target, and proposed that the selected aptamer could potentially be utilized in constructing novel imaging agents for HER2-positive cancers [20]. Kazem *et al.* also obtained a library of DNA aptamers by using HER2-positive cells as the target for aptamer selection [21]. So far, however, HER2 aptamer has not been explored as the tumor-targeting ligand for selective drug delivery to HER2-positive breast cancer cells. To investigate whether HER2 aptamer can be utilized in targeted HER2 therapy, we developed an aptamer-based doxorubicin delivery system in this study, and found that the aptamer could be employed to selectively deliver cytotoxic agent to HER2-postive breast cancer cells *in vitro*. The results suggest that, in addition to monoclonal antibodies, HER2 aptamers may also have application potentials in construction of novel HER2-targeted therapeutic systems.

There are multiple technical approaches to develop HER2 aptamers. One of them is termed Cell-SELEX, which uses the entire HER2-positive cell as the target during the aptamer selection process [37]. One potential drawback of this approach is that the selected aptamers may or may not bind to the HER2 structure, since these aptamers also may bind to the other proteins in the cell membrane of the target cell [30]. In order to select out aptamers that could truly bind to HER2 structure, here in this study, we chose an epitope sequence (peptide) of HER2 protein as the target in our SELEX process. The peptide sequence was a part of the extra-cellular domain (ECD) of HER2 protein, and contained 20 AAs. The advantage of this target peptide was that it could be synthesized with high purity, allowing us to select a population of aptamers that mostly bound to the target. Interestingly, some of the aptamers thus generated could also bind to the ECD of HER2 protein and the HER2-positive breast cancer cells (Figure 2&4). We postulated that this might be related to the properties of the peptide and the structure of the HER2 protein. First, the peptide is a confirmed B cell epitope that is found close to the cell membrane of the ECD of HER2 protein [38]. B cell epitope usually locates at a flexible and exposed part of the protein [30,39]. Second, HER2 crystal structure reveals that the extra-cellular domain of this protein is always in an extended conformation [40-43]. Thus, the target epitope is probably exposed on the surface of the HER2 protein, and the aptamer recognizing this epitope may consequently bind to the HER2 protein and the HER2-positive cells as well. *It is interesting that the K*_*d*_*for binding with the HER2 ECD protein was higher than that for binding with the HER2 peptide. One factor possibly contributing to the difference is that the original aptamer binding site on the HER2 peptide might be partially masked by the more complex structure of the HER2 ECD protein. It is also possible that the binding affinity of the aptamer might be weakened by the influences from the protein structures adjacent to the aptamer binding site.*

The binding preference of an aptamer is important for its application as a targeting ligand. An ideal tumor-targeting aptamer should bind to the target molecule (in this case, the HER2 structure), with minimal binding to other proteins. Since albumin is the most abundant protein in blood, we added BSA to the selection solution as a background component, in order to reduce the aptamer’s binding to albumin. As a result, the HER2 aptamer HB5 developed here had a weak binding to BSA, while maintaining a good binding profile to HER2 structures. Interestingly, the HER2 aptamer also had minimal reactivity to trypsin (Figure 2), which was commonly used for testing the binding specificity of the aptamers. Moreover, the HER2-aptamer exhibited a relatively strong binding to HER2-positive breast cancer cells, but minimal binding towards the HER2-negative cells (Figure 4). These results indicated that the HER2 aptamer HB5 exhibited certain degrees of targeting preference *in vitro*. Nevertheless, the aptamer’s *in vivo* HER2 preference still needs to be verified with future animal experiments in the follow-up studies.

Prior studies have shown that aptamers can deliver doxorubicin to tumor cells that bind the aptamer [28]. Similarly, in this study, the confocal microscopy demonstrated that the Apt-Dox selectively delivered doxorubicin to HER2-positive breast cancer cells (Figure 6C). Interestingly, in HER2-positive breast cancer cells, Apt-Dox stained both the nuclei and the cytoplasm, while the free Dox exclusively stained the nuclei. This observation may reflect a difference in the drug uptake mechanisms between Apt-Dox and the free Dox. We hypothesized that the lipophilic free Dox entered cells via passive diffusion, while the hydrophilic aptamer prevented the Apt-Dox from freely defusing into the lipid cell membrane. The Apt-Dox possibly entered HER2-positive cells via a receptor-mediated endocytosis. In other words, the aptamer in the Apt-Dox recognized and bound to the HER2 structure on HER2-positive cells. Since the binding site was very close to the cell membrane, the binding might induce a conformational change of HER2 protein and disturb the adjacent cell membrane, resulting in the endocytosis of the Apt-Dox complex. Obviously, extensive future research is needed to clearly unveil the mechanism by which the Apt-Dox entered the HER2-positive cancer cells. It should be noted that the Apt-Dox produced weak staining in the HER2-negatives cells, a finding consistent with the lack of HER2 expression in these cells (Figure 6D). The results indicated that the Apt-Dox had the capability to selectively deliver doxorubicin to HER2-positive breast cancer cells. This presumably would decrease the adverse effects of Dox against non-tumor tissues with low HER2 expression. Future research may concentrate on improving the binding kinetics, targeting efficacy, drug-loading capacity, and the *in vivo* stability of the targeted drug delivery system based on HER2 aptamers. In addition, extensive animal studies are also needed to evaluate the pharmacokinetics and the *in vivo* efficacy of the HER2-targeted drug-delivery system.

## Conclusions

In this study, a newly developed DNA aptamer (HB5) was found capable of binding to HER2 protein and HER2-positive *breast cancer* cells, with minimal binding to HER2-negative cells. A complex of the aptamer and doxorubicin (Apt-Dox) could selectively deliver doxorubicin to HER2-positive breast cancer cells while reducing the drug intake by HER2-negative cells. The results suggest that HER2 aptamers may have application potentials in targeted therapy against HER2-positive *breast cancer cells*.

## Competing interests

The authors declare that they have no competing interests.

## Authors’ contributions

Conceived and designed the experiments: XDY. Performed the experiments: ZL. Analyzed the data: XDY, ZL. Contributed reagents/materials/analysis tools: JHD, JM, YMS, FDW, XL. Wrote the paper: XDY, ZL. All authors read and approved the final manuscript.
